# Efficacy of Venom from Tentacle of Jellyfish *Stomolophus meleagris* (*Nemopilema nomurai*) against the Cotton Bollworm *Helicoverpa armigera*

**DOI:** 10.1155/2014/315853

**Published:** 2014-08-04

**Authors:** Huahua Yu, Rongfeng Li, Xiangli Dong, Ronge Xing, Song Liu, Pengcheng Li

**Affiliations:** ^1^Institute of Oceanology, Chinese Academy of Sciences, 7 Nanhai Road, Qingdao 266071, China; ^2^Qingdao Agricultural University, Chengyang, Qingdao 266109, China

## Abstract

Efficacy of venom from tentacle of jellyfish *Stomolophus meleagris* against the cotton bollworm *Helicoverpa armigera* was determined. Venom from tentacle of jellyfish *Stomolophus meleagris* could inhibit the growth of *Helicoverpa armigera* and the weight inhibiting rate of sample NFr-2 was 60.53%. Of the six samples, only NFr-2 had high insecticidal activity against *Helicoverpa armigera* and the corrected mortality recorded at 7 d was 74.23%.

*Helicoverpa armigera*, one of the most widely distributed noctuidae, occurs in Africa, Asia, Australia, Oceania, and Europe. The polyphagous larvae attack cotton, maize, sorghum, sunflower, tomato, okra, and a range of legumes, resulting in huge economic loss [1]. At present, the widely practised management programme to control the* Helicoverpa armigera* is chemical control, and the organophosphates, carbamates, pyrethroids, and other classes of insecticides have been used to control* Helicoverpa armigera*. However, significant insect resistance has emerged and residual agrochemicals in the environment have been becoming serious. In addition, transgenic crops, such as Bt cotton, are one of the successful methods to control this pest [2]. Nevertheless, the development of resistance to Bt is a major threat to the long-term use of toxins from* Bacillus thuringiensis* (Bt) in transgenic plants. So, development of novel, powerful, target-selective, and environment-friendly insecticides is necessary as an alternative approach to control bollworms.

Venomous animals produce diverse chemical cocktails that are used for defense, prey capture, competitor deterrence, and/or extraoral digestion [3]. These venoms have proved to be a valuable source of pharmacologically active compounds. The reports about the insecticidal activity of biological venom were mainly focused on the spider and scorpion venom [4–8]. The study on the insecticidal activity of venom from tentacle of jellyfish was deficient. Only our research group had reported that* Rhopilema esculentum* venom had different insecticidal activity against* Stephanitis pyri *Fabricius,* Aphis medicaginis *Koch, and* Myzus persicae *Sulzer and the 48 h LC_50_ values were 123.1 *μ*g/mL, 581.6 *μ*g/mL, and 716.3 *μ*g/mL, respectively [9].

*Stomolophus meleagris* L. Agassiz, 1862, also named* Nemopilema nomurai* Uchida, 1936, a cnidarian of the phylum Cnidaria, the class Scyphozoa, the order Semaeostomeae, the family Cyaneidae, and the genus* Stomolophus*, is distributed widely from the South China Sea and the Yellow Sea to the Bohai Sea and is abundant in late summer to early autumn [10]. Jellyfish venom from tentacle with unique structure has many bioactivities such as enzymatic activity, hemolysis, hepatocyte toxicity, cardiac toxicity, and antioxidant activity [11–16]. However, the insecticidal activity of venom from jellyfish* Stomolophus meleagris* had never been studied. In this study, efficacy of venom from tentacle of jellyfish* Stomolophus meleagris *against the cotton bollworm* Helicoverpa armigera* is assayed.

Jellyfish* Stomolophus meleagris* were collected in the Aoshan Bay in Qingdao, Shandong Province, China, in August 2012. Bloom of jellyfish is recently becoming more and more serious and seriously affects the tourism, fishing, military affairs, and marine sport events. So, fishing for jellyfish is permitted by the department of fisheries in China. Tentacles were manually excised in vivo, packed in polythene bags, and frozen immediately at −20°C. The frozen tentacles were then sonicated in cold (4°C) phosphate buffer solution (0.01 M, pH 6) eight times for 30 s each time at 100 mv. The resultant fluids were clarified by centrifugation (15,000 g) for 20 min at 4°C and used as full venom (SFV). The concentration was determined by the method of Bradford [17], using bovine serum albumin (BSA) as a standard. SFV was subjected to 30% (NH_4_)_2_SO_4_ saturation by adding solid (NH_4_)_2_SO_4_ with gentle stirring at 4°C. The mixture was left for 2 h for complete precipitation to occur and then centrifuged (15,000 g) for 20 min at 4°C. The precipitate was removed (Fr-1) and the supernatant was subjected to 60% (NH_4_)_2_SO_4_ saturation, and the whole process was repeated. Both precipitates (Fr-1 and Fr-2) were repeatedly dialysed in 0.01 M PBS (pH 6.0) to remove (NH_4_)_2_SO_4_. SFV, Fr-1, and Fr-2 were frozen at −20°C until use. NSFV, NFr-1, and NFr-2 were SFV, Fr-1, and Fr-2 stored at room temperature, respectively.

The diet incorporation assay was applied to determine the efficacy of the samples on the neonate larvae of* H. armigera*. SFV, Fr-1, Fr-2, NSFV, NFr-1, and N Fr-2 were added to the artificial diet and the concentrations of the samples were listed in Table 1. The neonate larvae of* H. armigera* were fed with the artificial feed that contained the samples. The mortality was recorded at 1 d, 3 d, and 7 d after the treatment and the weight of the larvae was checked at 7 d after the treatment. The neonate larvae fed with artificial feed without the samples were used as control. The mortality was recorded at 24 h and 48 h after the treatment. All data were expressed as means ±SD of three parallel measurements. Data were analyzed by Student's *t*-test and all tests were considered statistically significant at *P* < 0.05.

Table 1 shows the results of the samples against the neonate larvae of* H. armigera*. Of the six samples, NFr-2 had the strongest insecticidal activity against the neonate larvae of* H. armigera* and the corrected mortality recorded at 7 d of NFr-2 was 74.23%. SFV, Fr-1, Fr-2, NSFV, and NFr-1 had low toxicity on the neonate larvae of* H. armigera* and the corrected mortality was below 8%. From the results of weight recorded at 7 d, NSFV, NFr-1, and NFr-2 inhibited the growth of neonate larvae. The weight inhibiting rates were 38.07–60.53%. Fr-2 also inhibited the growth of neonate larvae, but the maximal weight inhibiting rate was 19.06%. For SFV and Fr-1, at the concentrations 1.93 *μ*g/g and 0.6 *μ*g/g, respectively, the growth of larvae was facilitated, while at the concentrations 19.3 *μ*g/g and 6 *μ*g/g, respectively, the growth of larvae was inhibited and the weight inhibiting rate was 8.05% and 6.54%, respectively.

A multitude of substances with bioactivity against* H. armigera *have already been isolated from plant and bacteria materials [18–22]. Zhao et al. had reported on the growth and development of* H. armigera* affected by the extracts of the twelve Chinese medicines. Ten Chinese medicines (*Brucea javanica*,* Nerium indicum*,* Scutellaria barbata*,* Melia azedarach*,* Brassica alba*,* Rheum palmatum*, Herbal* Andrographis*,* Ulmus macrocarpa*,* Toona sinensis*, and* Folium isatidis*) had inhibited the growth of* H. armigera* and the weight inhibiting rates recorded at 5 d were 17.25–102.19%, but two Chinese medicines* Semen Pharbitidis and Bitter Almond* had facilitated the growth of* H. armigera *[23]. NSFV, NFr-1, and NFr-2 had higher growth inhibiting rate than the extract of* Folium isatidis*. Feng et al. had studied the effect of insecticidal plant extracts on* Heliothis armigera*. The results showed that ten plant extracts (*Karelinia caspica *Cess,* G. inflata *Batal,* Chenopodium glaucum* L.,* Convolvulus arvensis* L.,* P. hendersonii *Woodsnn,* Apocynum venetum *L.,* Datum stramonium *L.,* Inula salsoloides *(Turcz) Ostenf,* Descurainia sophia *(L.) Schur, and* Scorzonera divaricata *Turcz) inhibited the growth of* H. armigera* and eleven plant extracts (*L. ruthenicum *Murr.,* Karelinia caspica *Cess,* G. inflata *Batal,* Chenopodium glaucum* L.*, Convolvulus arvensis* L.*, P. hendersonii *Woodsnn,* Apocynum venetum *L.,* Datum stramonium *L.,* Inula salsoloides *(Turcz) Ostenf,* Descurainia sophia *(L.) Schur, and* Scorzonera divaricata *Turcz) had insecticidal activity against* H. armigera*, but the weight inhibiting rates and the corrected mortality were low. The max weight inhibiting rate and corrected mortality were 36.32% and 31.20%, respectively [24]. NSFV, NFr-1, and NFr-2 had higher growth inhibiting rates than these ten plant extracts. NFr-2 had higher insecticidal activity against* H. armigera *than these eleven plant extracts. In addition, Zhu et al. reported that Bt reduced body weight by 31%, below the growth inhibiting rate of NSFV, NFr-1, and NFr-2 [25].

SFV, NFr-1, and NFr-2 inhibited the growth of* H. armigera*. It was speculated that this phenomenon was probably in connection with the metabolizable capability of* H. armigera*. By enhancing the activity of metabolizable enzymes,* H. armigera* conquered the maladjustment of normal metabolizable balance that resulted from NSFV, NFr-1, and NFr-2. So,* H. armigera* consumed much energy and amino acid for the synthesis of digestive enzymes. Consequently, amino acid proportion was out of balance and* H. armigera* suffered from malnutrition and weight loss. The larvae period was delayed by the inhibiting effect on growth, which could alleviate the dangerous level of pest on the growth and increase the sensitivity of individual larva against other lethal factors. Thus more far-reaching control impact brought from the inhibiting effect was obtained than that of the insecticidal activity.

SFV, Fr-1, Fr-2, NRFV, and NFr-1 had no or low insecticidal activity against the neonate larvae of* H. armigera*. Possible reasons were that* H. armigera* was not the target pest of the test samples and the concentration of samples was too low to kill most* H. armigera*. The corrected mortality recorded at 7 d of NFr-2 was 74.23%, but according to the symptom, the death might be due to disease resulting from fungus or bacterium. Some funguses or bacteria were mighty cultured by NFr-2 and the metabolites of these funguses or bacteria had insecticidal activity against the neonate larvae of* H. armigera*. Jung et al. isolated four new pyrazinoquinazoline indole glucosides from the fungus* Aspergillus fumigatus *and four new cytochalasin derivatives and cytochalasin B from the fungus* Phoma *sp. obtained from the jellyfish* Nemopilema nomurai *[26, 27]. Wright et al. isolated one epicoccamide from the fungus* Epicoccum purpurascens *obtained from the inner tissue of the jellyfish* Aurelia aurita *[28]. However, the insecticidal activity of the metabolites from fungus obtained from jellyfish was not studied. So, culturing and separating the fungus and actinomyces with high insecticidal activity from jellyfish is necessary to be further studied.

In conclusion, venom from tentacle of jellyfish* Stomolophus meleagris* could inhibit the growth of* Helicoverpa armigera*. The samples NSFV, NFr-1, and NFr-2 stored at room temperature had higher weight inhibiting rate than those of SFV, Fr-1, and Fr-2 stored at −20°C. NFr-2 had insecticidal activity against* Helicoverpa armigera *and the corrected mortality was up to 74.23%.

## Figures and Tables

**Table 1:** Results of the samples against the neonate larvae of *H. armigera*.

Samples	Concentration (*μ*g/g)	1 d	3 d	7 d		
		Corrected mortality (%)	Corrected mortality (%)	Corrected mortality (%)	Weight (mg)	Weight inhibiting rate (%)
Control	0	—	—	—	17.26 ± 0.66	
SFV	1.93	0	0	0	19.53 ± 0.60	−13.15 ± 0.34
	19.29	0	0	0	15.87 ± 1.12	8.05 ± 2.66
Fr-1	0.6	0	0	1.12 ± 0.01	20.73 ± 2.02	−20.10 ± 7.88
	6	0	1.12 ± 0.01	1.12 ± 0.01	16.13 ± 1.81	6.54 ± 6.66
Fr-2	0.64	0	0	0	15.5 ± 1.49	10.20 ± 4.81
	12.81	0	0	0	13.97 ± 0.79	19.06 ± 1.79

Control	0	—	—	—	35.17 ± 1.76	
NSFV	1.39	12.3 ± 0.05	15.27 ± 0.07	7.04 ± 0.07	19.01 ± 2.98	45.95 ± 3.47
	13.93	9.94 ± 0.12	11.05 ± 0.14	4.53 ± 0.08	17.87 ± 1.41	49.19 ± 1.00
NFr-1	0.44	1.85 ± 0.06	3.71 ± 0.05	0.78 ± 0.02	18.18 ± 1.48	48.31 ± 0.80
	4.37	1.75 ± 0.12	1.75 ± 0.11	4.45 ± 0.10	15.52 ± 0.40	55.87 ± 3.87
NFr-2	1.41	0	3.71 ± 0.09	67.65 ± 1.03	21.78 ± 0.88	38.07 ± 2.50
	14.07	0	2.22 ± 0.08	74.23 ± 1.86	13.88 ± 0.41	60.53 ± 3.84
